# Comparison of bilateral differential characteristics of corneal biomechanics between keratoconus and normal eyes

**DOI:** 10.3389/fbioe.2023.1163223

**Published:** 2023-06-01

**Authors:** Yiyong Xian, Yu Zhao, Ling Sun, Xiaoyu Zhang, Lan Ding, Zesheng Liu, Yuan Li, Yanlan Ding, Lin Jiang, Xingtao Zhou, Yang Shen

**Affiliations:** ^1^ Department of Ophthalmology and Optometry, Eye and ENT Hospital, Fudan University, Shanghai, China; ^2^ NHC Key Laboratory of Myopia, Key Laboratory of Myopia, Fudan University, Chinese Academy of Medical Sciences, Shanghai, China; ^3^ Shanghai Research Center of Ophthalmology and Optometry, Shanghai, China; ^4^ Shanghai Engineering Research Center of Laser and Autostereoscopic 3D for Vision Care (20DZ2255000), Shanghai, China; ^5^ Shangqiu First People’s Hospital, Shangqiu, China

**Keywords:** keratoconus, corneal ectasia, biomechanics, forme fruste keratoconus, topography, tomography

## Abstract

**Purpose:** To compare bilateral differences in corneal biomechanics between keratoconus and normal eyes.

**Methods:** In this case-control study, 346 eyes of 173 patients (aged 22.1 ± 6.1 years) with keratoconus (KC group) and 378 eyes of 189 patients (aged 26.7 ± 5.6 years) with ametropia (control group) were enrolled. Corneal tomography and biomechanical properties were examined using Pentacam HR and Corvis ST, respectively. The corneal biomechanical parameters were compared between eyes with forme fruste keratoconus (FFKC) and normal eyes. Bilateral differences in corneal biomechanical parameters were compared between the KC and control groups. Receiver operating characteristic (ROC) analysis was used to assess discriminative efficacies.

**Results:** The areas under the ROC curves (AUROCs) of stiffness parameter at the first applanation (SP-A1) and Tomographic and Biomechanical Index (TBI) for identifying FFKC were 0.641 and 0.694, respectively. The bilateral differential values of major corneal biomechanical parameters were significantly increased in the KC group (all *p* < 0.05), except for the Corvis Biomechanical Index (CBI). The AUROCs of the bilateral differential values of the deformation amplitude ratio at 2 mm (ΔDAR2), Integrated Radius (ΔIR), SP-A1 (ΔSP-A1), and the maximum inverse concave radius (ΔMax ICR) for discriminating keratoconus were 0.889, 0.884, 0.826, and 0.805, respectively. The Logistic Regression Model-1 (comprising of ΔDAR2, ΔIR, and age) and the Logistic Regression Model-2 (comprising of ΔIR, ΔARTh, ΔBAD-D, and age) had AUROCs of 0.922 and 0.998, respectively, for discriminating keratoconus.

**Conclusion:** The bilateral asymmetry of corneal biomechanics was significantly increased in keratoconus compared with normal eyes, which may be helpful for the early detection of keratoconus.

## Introduction

Keratoconus (KC) is a primary corneal ectatic disease characterized by progressive thinning and conic protrusion of the cornea and has an incidence rate of approximately 1/2000 (Gomes et al., 2015). The corneal morphology of advanced keratoconus is significantly altered and has an irregular pattern, which results in complex refractive errors that are difficult to correct with spectacles and thus severely affect patients’ visual function and quality of life (Kandel et al., 2022) and cause heavy socioeconomic burdens (Rebenitsch et al., 2011).

Currently, it is not a challenge to diagnose clinical and subclinical keratoconus with modern corneal tomographers and assessment of corneal biomechanics (Vinciguerra et al., 2016; Ambrosio et al., 2017). However, the standards of corneal morphology and biomechanics for KC screening before corneal refractive surgeries are more stringent, and ophthalmologists aim to identify early keratoconus or high-risk corneas to avoid or reduce the risk of postoperative corneal ectasia (Xie et al., 2020). However, the corneal tomography of early keratoconus, especially forme fruste keratoconus (FFKC), may have no abnormalities, and the corneal biomechanics may also be normal. The diagnosis can only be made based on the contralateral eye being clinical keratoconus.

Bilateral asymmetry is a key feature of keratoconus (Gomes et al., 2015) and researchers have proven that the onset of keratoconus differed for each eye (Zadnik et al., 2002; Nichols, 2004). It is reasonable to believe that an abnormal increase in the inter-eye asymmetry of corneal morphology or biomechanics occurs before the more severe eye in patients with FFKC reaches the clinical stage. Diagnostic models for keratoconus, such as the Belin/Ambrosio Enhanced Ectasia Display and the total deviation value (BAD-D), the Corvis Biomechanical Index (CBI), and the Tomographic and biomechanical index (TBI), only judge on the examination data of one eye (Vinciguerra et al., 2016; Ambrosio et al., 2017), ignoring the information of bilateral asymmetry. Our previous study adopted a novel algorithm to quantitively compare the bilateral asymmetry of corneal tomography between keratoconus and normal eyes. The area under the receiver operating characteristic curves (AUROC) for screening keratoconus (including FFKC) reached 0.985 (Shen et al., 2021), significantly higher than that of the traditional CBI and TBI models.

Reportedly, the loss of biomechanical stability in keratoconus was considered to happen earlier than morphologic changes and induce a cyclic cascade leading to thinning and bulging of the cornea (Roberts and Dupps, 2014). Therefore, this study aimed to investigate the bilateral asymmetry of corneal biomechanics between keratoconus (including FFKC) and normal eyes and to evaluate the clinical value of bilateral asymmetry of biomechanical parameters for the early detection of keratoconus.

## Subjects and methods

This case-control study was conducted in accordance with the principles of the Declaration of Helsinki and was approved by the Ethics Committee of the Eye and ENT Hospital of Fudan University (2022049). Informed consent was obtained from all patients included in the study.

Patients diagnosed with keratoconus, based on the Global Consensus on Keratoconus Diagnosis from 2015 by experienced experts (XTZ and YS), were included and were allocated to the KC group (*n* = 173, including 108 patients with bilateral clinical keratoconus, 21 patients with clinical keratoconus in one eye and subclinical keratoconus in the contralateral eye, and 44 patients with clinical keratoconus in one eye with forme fruste keratoconus in the contralateral eye). Within the KC group, the FFKC in one eye (*n* = 44) was diagnosed based on the presence of manifest keratoconus in the contralateral eye when the eye itself was asymptomatic on slit lamp, topographic (paracentral inferior-superior dioptric asymmetry ≤1.4), and tomographic (central anterior and posterior elevations <8 and 13 μm, respectively, with the Best-Fit-Sphere [BFS] as the reference sphere) examinations (Ruiz Hidalgo et al., 2016; Awad et al., 2017). Patients who had undergone corneal refractive surgery for myopia and had stable conditions for 2 years postoperatively were included in the control group (*n* = 189), and the preoperative examination records of these patients were used in this study.

Before the examinations, the patients were asked to discontinue the use of soft contact lenses or rigid gas-permeable contact lenses for 2 or 4 weeks, respectively. Patients with a history of ophthalmic surgery, trauma, other eye diseases (e.g., glaucoma), systemic diseases (e.g., connective tissue disease), or serious psychological or psychiatric diseases were excluded from the study.

**FIGURE 1:**
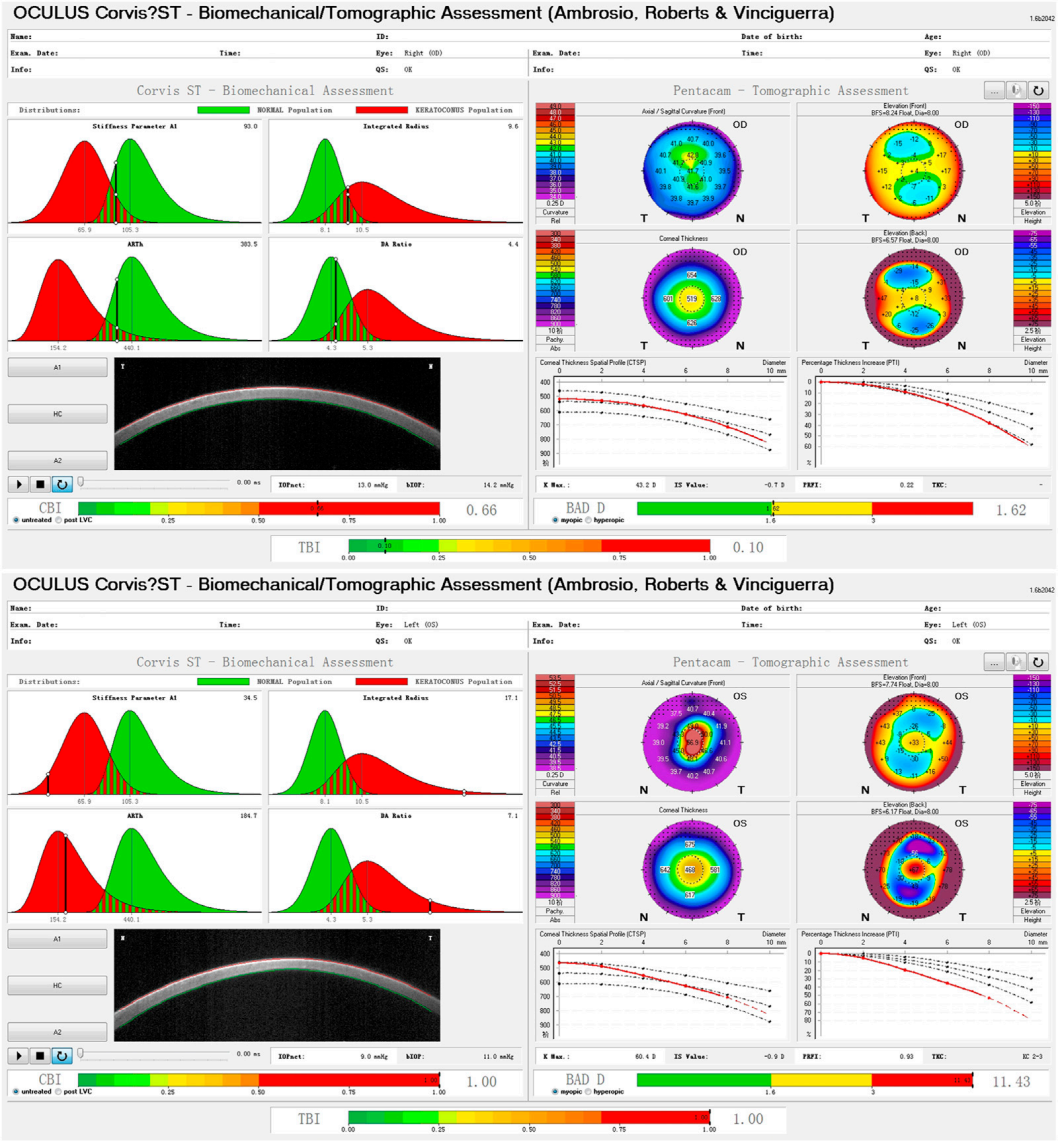
Bilateral differences of corneal biomechanics and tomography in a patient with keratoconus.

A total of 346 eyes from 173 patients were included in the KC group, with an average age of 22.1 ± 6.1 years, while the control group comprised 378 eyes of 189 patients, with an average age of 26.7 ± 5.6 years (*Z* = −2.236, *p* = 0.025, Mann-Whitney *U* test). Figure 1 shows the examination results of bilateral corneal tomography and biomechanics from the same patient with keratoconus as an example and significant bilateral differences were displayed.

### Examinations

1) Corneal tomography was conducted using the Pentacam HR (Oculus Optikgerate GmbH, Wetzlar, Germany) Scheimpflug-based anterior segment analyzer according to standard operating procedures (Shen et al., 2019). A total of 25 images were captured in 2 s by the Pentacam HR system using a 475 nm monochromatic blue light-emitting diode and a Scheimpflug camera that rotated around the corneal axis (Kreps et al., 2020). Parameters including mean or maximal keratometry of the anterior surface (FKm/FKmax), central corneal thickness (CCT), thinnest corneal thickness (TCT), and the BAD-D (the total deviation value in the Belin/Ambrosio Enhanced Ectasia display [BAD] system) were recorded. 2) The corneal biomechanical characteristics were analyzed using the Corvis ST (Oculus Optikgerate GmbH, Wetzlar, Germany) non-contact Scheimpflug-based tonometer according to the standard operating procedures by automatic release (Shen et al., 2019), and major biomechanical parameters were recorded as described in the next section. One examination was taken per eye by experienced technicians.

### Definitions of the main corneal tomographic and biomechanical parameters

The Corvis ST uses a high-speed Scheimpflug camera to capture the deformation process of the cornea at the horizontal meridian after being subjected to an air puff. The time when the camera starts is set as zero, and the instrument automatically analyzes and obtains three landmark time points during the deformation process: 1) the first applanation (A1): A1 is the moment when the cornea begins to change from a convex shape to a concave shape, and the central cornea is compressed and flattened for the first time; 2) the highest concavity (HC): HC is the moment when the central cornea undergoes the greatest degree of deformation and concavity due to an air puff; and 3) the second applanation (A2): A2 is the second flattening moment of the cornea when it is restored from the highest concavity. (Vellara and Patel, 2015).

As shown in Table 1, major parameters derived from the Corvis ST were included in the study: 1) A1V/A2V: vertical velocity of the corneal apex at A1 or A2; 2) A1DeflA/A2DeflA: vertical deflection amplitude of the corneal apex at A1 or A2; 3) SP-A1: the ratio of the actual pressure on the cornea to the corresponding deflection amplitude at A1; 4) DeflA Max: vertical deflection amplitude of the corneal apex at HC; 5) DAR2/DAR1: the ratio of the deformation amplitude of the central cornea and cornea at paracentral 2mm/1 mm at HC; 6) Max ICR and IR: a curve describing the relationship between time and inverse radius (1/R) of the cornea can be depicted during deformation, through which the maximal inverse concave radius (Max ICR) and the area under the curve (Integrated Radius [IR]) can be calculated; 7) ARTh: the ratio of the thinnest pachymetry at the horizontal meridian to the pachymetric progression index (Vinciguerra et al., 2016); 8) CBI: The Corvis biomechanical index (CBI) is based on logistic regression analysis using the corneal thickness profile and deformation parameters (Vinciguerra et al., 2016); and 9) SSI: The stress-strain index (SSI) reflects the material stiffness of corneas *in vivo,* which would decrease in keratoconus (Eliasy et al., 2019).

**TABLE 1:** Abbreviations and definitions of major corneal biomechanical and tomographic parameters.

Abbreviations	Definitions
A1V	the velocity at the first applanation
A2V	the velocity at the second applanation
A1DeflA	the deflection amplitude at the first applanation
A2DeflA	the deflection amplitude at the second applanation
DeflA Max	the maximum deflection amplitude
Max ICR	the maximum inverse concave radius
DAR2	the deformation amplitude ratio at 2 mm
DAR1	the deformation amplitude ratio at 1 mm
ARTh	the Ambrosio Relational Thickness horizontal
IR	Integrated Radius
SP-A1	the stiffness parameter at the first applanation
CBI	the Corvis Biomechanical Index
TBI	the Tomographic and Biomechanical Index
SSI	the stress-strain index
BAD-D	the Belin/Ambrosio Enhanced Ectasia Deviation value

The Tomographic and Biomechanical Index (TBI) is a combined tomographic and biomechanical parameter derived from analyzing the data from the Pentacam and Corvis ST and is used for detecting keratoconus (Ambrosio et al., 2017).

### Statistical analysis

Statistical analyses were performed using the Statistical Package for the Social Sciences (version 24.0, SPSS, Inc., Chicago, IL, United States) and MedCalc Statistical Software version 20 (MedCalc Software Ltd., Ostend, Belgium).

The right eyes of patients with bilateral clinical keratoconus and the eyes with subclinical/forme fruste keratoconus of patients with very asymmetric keratoconus were selected to form a group to compare with the right eyes of patients in the control group in terms of biomechanical and tomographic properties. For comparison between the FFKC and control eyes, 44 right eyes from the control group were randomly selected.

The bilateral differential parameters were calculated by the absolute differences in binocular parameters and were represented by adding a “Δ” before the name of the corresponding parameters. For example, the bilateral differential parameter “ΔA1V,” defined as “binocular asymmetry of A1V,” was calculated as the absolute difference between the binocular A1V values.

Continuous variables are described as mean ± standard deviation and 95% confidence intervals (CI), and categorical variables are expressed as frequencies. The Shapiro-Wilk test was used as the normality test. As most parameters were non-normally distributed, the Mann-Whitney *U* test was used to test the difference between the two independent groups. Eyes with greater maximal keratometry of the anterior surface (FKmax) in keratoconus were defined as the “worse eye” and the corresponding FKmax was represented as “FKmax (worse).” Pearson correlation analysis was used to analyze the correlation between FKmax (worse) and the other parameters. Logistic regression analysis was used to construct multivariable models for discriminating keratoconus, and the forward stepwise (conditional) method, a built-in method in the SPSS software, was used for feature selection. The receiver operating characteristic (ROC) analysis was used to evaluate the diagnostic performance of the parameters or models, and the area under the ROC curves (AUROC) and 95% CI were calculated. The optimal cutoff was determined using the maximal Youden index (sensitivity + specificity – 1), and the corresponding sensitivity and specificity were reported. The DeLong test (DeLong et al., 1988) was used to compare the AUROCs between the parameters. All data were examined by a two-tailed test, and *p* < 0.05 was considered statistically significant.

## Results

### Demographics

In the KC group, the mean keratometry of the anterior surface (FKm) was 47.4 ± 5.2 D, and the TCT was 477 ± 46 μm, while FKm and TCT in the Control group were 43.5 ± 1.3 D and 538 ± 27 μm, respectively (both *p* < 0.001). The BAD-D, CBI, and TBI values were significantly different between the two groups (all *p* < 0.001). The other demographic data are presented in Table 2.

**TABLE 2:** Main demographic data of the keratoconus and control groups.

Parameters	KC group (*n* = 346)	Control group (*n* = 378)	*Z* ^a^	*p* ^a^
	Mean ± SD (95% CI)	Mean ± SD (95% CI)		
FKm (D)	46.5 ± 5.3 (45.7, 47.3)	43.5 ± 1.3 (43.4, 43.7)	−9.191	<0.001
FKmax (D)	52.7 ± 9.8 (51.2, 54.1)	44.7 ± 1.5 (44.5, 44.9)	−10.488	<0.001
CCT (μm)	491 ± 48 (483, 498)	541 ± 27 (537, 545)	−8.962	<0.001
TCT (μm)	484 ± 49 (477, 491)	538 ± 27 (534, 542)	−8.999	<0.001
BAD-D	6.43 ± 5.5 (5.60, 7.26)	1.21 ± 0.5 (1.14, 1.28)	−10.522	<0.001
ARTh	321.7 ± 153.7 (298.7, 344.8)	493.6 ± 92 (480.4, 506.8)	−9.096	<0.001
SP-A1 (mmHg/mm)	79.8 ± 24.3 (76.2, 83.5)	110 ± 15.7 (107.8, 112.3)	−7.983	<0.001
CBI	0.67 ± 0.43 (0.61, 0.74)	0.12 ± 0.22 (0.09, 0.16)	−9.454	<0.001
TBI	0.83 ± 0.31 (0.78, 0.88)	0.23 ± 0.17 (0.20, 0.25)	−10.538	<0.001

Notes: KC, keratoconus; SD, standard deviation; 95% CI, 95% confidence interval for the mean; FKm, mean keratometry of the anterior surface; FKmax, maximum keratometry of the anterior surface; CCT, central corneal thickness; TCT, the thinnest corneal thickness; BAD-D, the Belin/Ambrosio Enhanced Ectasia Deviation value; ARTh, the Ambrosio Relational Thickness horizontal; SP-A1, stiffness parameter at the first applanation; CBI, corvis biomechanical index; TBI, tomographic and biomechanical index; D, diopter; μm, micron; and mmHg/mm, millimeter of mercury per millimeter.

^a^ Mann-Whitney *U* test.

### Discriminative efficacies of main biomechanical and tomographic parameters

The efficacies of the main biomechanical and tomographic parameters for discriminating KC are presented in Supplementary Table S1. The TBI, BAD-D, SP-A1, CBI, and ARTh had good discriminative ability for KC, with AUROC of 0.905, 0.900, 0.851, 0.823, and 0.818, respectively (all *p* < 0.001). Supplementary Table S2 shows the differences and discriminative ability of corneal biomechanical parameters between the FFKC and control groups. While other parameters were not significantly different between the two groups, TBI was significantly increased (all *p* < 0.01) and SP-A1 was significantly decreased (*p* = 0.023) in the FFKC group. The AUROCs of TBI and SP-A1 for discriminating FFKC were 0.694 and 0.641, respectively (all *p* < 0.05). The BAD-D was significantly increased in the FFKC group (*p* = 0.006), and the AUROC was 0.670 for identifying FFKC. The ROC curves of the major biomechanical and tomographic parameters for discriminating FFKC are shown in Supplementary Figure S1.

### Characteristics of bilateral differences of corneal biomechanical parameters

Table 3 displays the bilateral differences in biomechanical parameters between the two groups. Except for ΔCBI, the other bilateral differential parameters were significantly higher in the KC group than in the control group (all *p* < 0.01). Pearson correlation analysis between FKmax (worse) and bilateral differential parameters (Table 3) showed that FKmax (worse) had the highest positive correlation with ΔDAR2 (*r* = 0.606, *p* < 0.001) (Supplementary Figure S2B) and a mild positive correlation with ΔMax ICR, ΔIR, and ΔBAD-D (*r* = 0.438, 0.481, and 0.574, respectively; all *p* < 0.001) (Supplementary Figures S2A, S2E, S2J). Weak but significant correlations were found between FKmax (worse) and ΔDAR1, ΔSP-A1, ΔCBI, ΔTBI, and ΔSSI (all |r| <0.4, *p* < 0.01) (Supplementary Figures S2C, S2F–I). No significant correlation was found between FKmax (worse) and ΔARTh (*r* = 0.007, *p* = 0.924) (Supplementary Figure S2D). The relationship between FKmax (worse) and major bilateral differential parameters is shown in Supplementary Figure S2.

**TABLE 3:** Differences of parameters of bilateral corneal biomechanics and the correlation with FKmax of the worse eye.

Parameters	KC group (*n* = 173)	Control group (*n* = 189)	*Z* ^a^	*P* ^a^	Correlations with FK_max_ (worse)	
	Mean ± SD (95% CI)	Mean ± SD (95% CI)			*r*	*p*
ΔA1V (m/s)	0.02 ± 0.02 (0.02, 0.03)	0.01 ± 0.01 (0.01, 0.01)	−7.218	<0.001	0.413	<0.001
ΔA2V (m/s)	0.04 ± 0.03 (0.04, 0.05)	0.02 ± 0.01 (0.02, 0.02)	−8.112	<0.001	0.436	<0.001
ΔA1DeflA (mm)	0.02 ± 0.01 (0.01, 0.02)	0 ± 0 (0, 0.01)	−9.190	<0.001	0.598	<0.001
ΔA2DeflA (mm)	0.02 ± 0.02 (0.02, 0.02)	0.01 ± 0.01 (0.01, 0.01)	−6.927	<0.001	0.439	<0.001
ΔDeflA Max (mm)	0.11 ± 0.09 (0.10, 0.12)	0.04 ± 0.03 (0.04, 0.05)	−8.882	<0.001	0.474	<0.001
ΔMax ICR (mm^−1^)	0.04 ± 0.04 (0.04, 0.05)	0.01 ± 0.02 (0.01, 0.02)	−10.045	<0.001	0.438	<0.001
ΔDAR2	1.28 ± 1.1 (1.12, 1.45)	0.19 ± 0.22 (0.16, 0.22)	−12.784	<0.001	0.606	<0.001
ΔDAR1	0.08 ± 0.07 (0.07, 0.09)	0.02 ± 0.02 (0.02, 0.03)	−9.700	<0.001	0.316	<0.001
ΔARTh	145.93 ± 115.14 (128.65, 163.21)	51.93 ± 49.21 (44.87, 58.99)	−9.214	<0.001	0.007	0.924
ΔMax ICR (mm^−1^)	2.69 ± 2.12 (2.37, 3.00)	0.49 ± 0.35 (0.44, 0.54)	−12.609	<0.001	0.481	<0.001
ΔSP-A1 (mmHg/mm)	21.49 ± 14.75 (19.28, 23.71)	7.36 ± 6.38 (6.44, 8.28)	−10.715	<0.001	0.368	<0.001
ΔCBI	0.3 ± 0.4 (0.24, 0.36)	0.11 ± 0.17 (0.08, 0.13)	−1.037	0.300	−0.230	0.002
ΔTBI	0.16 ± 0.31 (0.11, 0.21)	0.12 ± 0.11 (0.10, 0.13)	−8.359	<0.001	−0.209	0.006
ΔSSI	0.18 ± 0.15 (0.16, 0.20)	0.07 ± 0.06 (0.06, 0.08)	−8.764	<0.001	0.224	0.003
ΔBAD-D	7.53 ± 5.31 (6.73, 8.32)	1.21 ± 0.5 (1.14, 1.28)	−15.055	<0.001	0.574	<0.001

Notes: KC, keratoconus; SD, standard deviation; 95% CI, 95% confidence interval for mean; ΔA1V, asymmetry of velocity at the first applanation; ΔA2V, asymmetry of velocity at the second applanation; ΔA1DeflA, asymmetry of the deflection amplitude at the first applanation; ΔA2DeflA, asymmetry of the deflection amplitude at the second applanation; ΔDeflA Max, asymmetry of the maximum deflection amplitude; ΔMax ICR, asymmetry of the maximum inverse concave radius; ΔDAR2, asymmetry of the deformation amplitude ratio at 2mm; ΔDAR1, asymmetry of the deformation amplitude ratio at 1mm; ΔARTh, asymmetry of the Ambrosio Relational Thickness horizontal; ΔIR, asymmetry of the integrated radius; ΔSP-A1, asymmetry of the stiffness parameter at the first applanation; ΔCBI, asymmetry of the Corvis Biomechanical Index; ΔTBI, asymmetry of the Tomographic and Biomechanical Index; ΔSSI, asymmetry of the stress-strain index; ΔBAD-D, asymmetry of the Belin/Ambrosio Enhanced Ectasia Deviation value; mmHg/mm, millimeter of mercury per millimeter; mm, millimeter; ms, millisecond; and m/s, meter per second.

^a^ Mann-Whitney *U* test.

### ROC analysis

**FIGURE 2:**
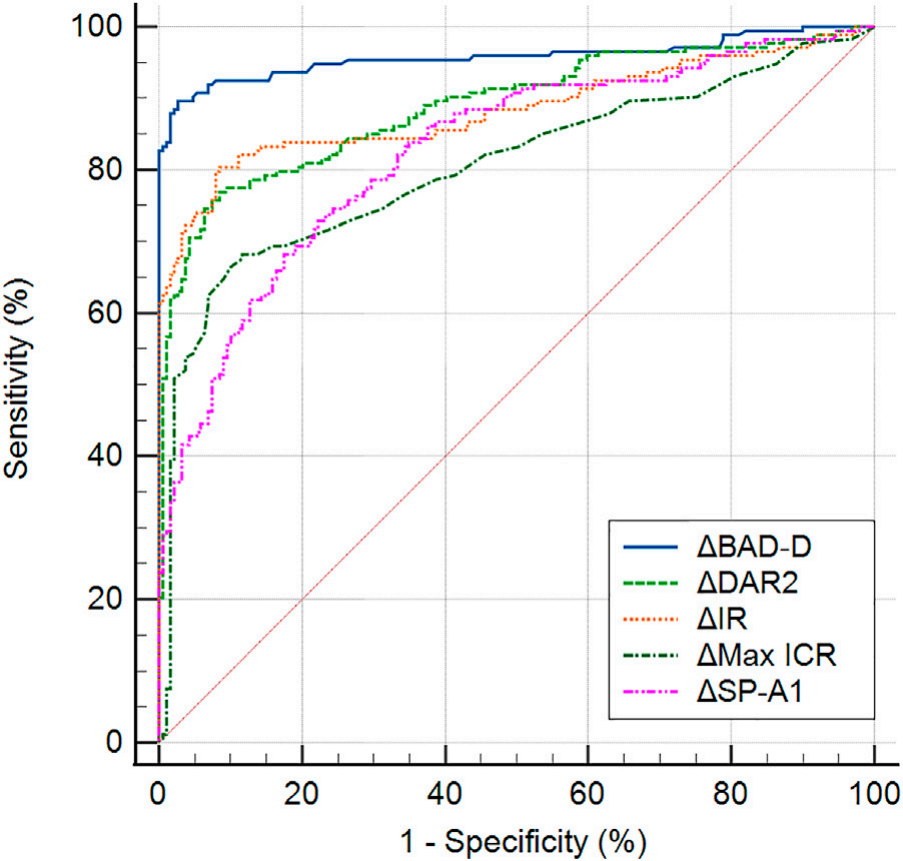
Receiver operating characteristic curves of major bilateral differential parameters, ΔDAR2 (asymmetry of the deformation amplitude ratio at 2 mm), ΔIR (asymmetry of Integrated Radius), ΔMax ICR (asymmetry of the maximum inverse concave radius), ΔSP-A1 (asymmetry of the stiffness parameter at the first applanation), and ΔBAD-D (asymmetry of the Belin/Ambrosio Enhanced Ectasia Deviation value), for keratoconus *versus* the control group.

Table 4 presents the ROC analysis of the bilateral differential biomechanical parameters and Figure 2 shows the ROC curves of the major parameters. The accuracy of the ΔDAR2, ΔIR, ΔSP-A1, and ΔMax ICR was good for discriminating KC, with AUROCs of 0.889, 0.884, 0.826, and 0.805, respectively (all *p* < 0.001) (Figure 2). When the cutoff was 0.41, the sensitivity and specificity of the ΔDAR2 were 76.9% and 91.5%, respectively. The sensitivity and specificity of ΔIR were 80.3% and 91.5%, respectively, with a cutoff of 0.93 mm^−1^.

**TABLE 4:** Receiver operating characteristics analysis for parameters of bilateral corneal biomechanics and tomography.

Parameters	AUROC	Sig.	95% CI	Cut-off	Sensitivity (%)	Specificity (%)
ΔA1V (m/s)	0.719	<0.001	0.666 to 0.773	0.02	53.8	83.6
ΔA2V (m/s)	0.747	<0.001	0.695 to 0.798	0.03	57.2	86.8
ΔA1DeflA (mm)	0.779	<0.001	0.731 to 0.827	0.01	62.4	81.5
ΔA2DeflA (mm)	0.710	<0.001	0.656 to 0.765	0.02	49.7	87.3
ΔDeflA Max (mm)	0.770	<0.001	0.721 to 0.819	0.09	53.2	91.0
ΔMax ICR (mm^−1^)	0.805	<0.001	0.758 to 0.853	0.02	61.5	89.8
ΔDAR2	0.889	<0.001	0.854 to 0.924	0.41	76.9	91.5
ΔDAR1	0.795	<0.001	0.747 to 0.843	0.05	59.1	90.5
ΔARTh	0.780	<0.001	0.732 to 0.829	100.47	57.8	88.9
ΔIR (mm^-1^)	0.884	<0.001	0.845 to 0.922	0.93	80.3	91.5
ΔSP-A1 (mmHg/mm)	0.826	<0.001	0.783 to 0.869	12.90	68.2	82.5
ΔCBI	0.531	0.138	0.468 to 0.595	0.50	30.6	95.8
ΔTBI	0.750	<0.001	0.688 to 0.812	0.01^a^	71.7	94.2
ΔSSI	0.767	<0.001	0.717 to 0.816	0.11	61.8	83.1
ΔBAD-D	0.958	<0.001	0.934 to 0.982	2.12	89.6	97.4
LRM-1	0.922	<0.001	0.893 to 0.952	0.54	78.6	95.8
LRM-2	0.998	<0.001	0.996 to 1.000	0.37	98.8	97.9

Notes: AUROC, area under the curves of receiver operating characteristic; Sig, significance; 95% CI, 95% confidence interval for AUROC; Cut-off, the threshold values; ΔA1V, asymmetry of velocity at the first applanation; ΔA2V, asymmetry of velocity at the second applanation; ΔA1DeflA, asymmetry of the deflection amplitude at the first applanation; ΔA2DeflA, asymmetry of the deflection amplitude at the second applanation; ΔDeflA Max, asymmetry of the maximum deflection amplitude; ΔMax ICR, asymmetry of the maximum inverse concave radius; ΔDAR2, asymmetry of the deformation amplitude ratio at 2 mm; ΔDAR1, asymmetry of the deformation amplitude ratio at 1 mm; ΔARTh, asymmetry of the Ambrosio Relational Thickness horizontal; ΔIR, asymmetry of the integrated radius; ΔSP-A1, asymmetry of the stiffness parameter at the first applanation; ΔCBI, asymmetry of the Corvis Biomechanical Index; ΔTBI, asymmetry of the Tomographic and Biomechanical Index; ΔSSI, asymmetry of the stress-strain index; ΔBAD-D, asymmetry of the Belin/Ambrosio Enhanced Ectasia Deviation value; mmHg/mm, millimeter of mercury per millimeter; mm, millimeter; ms, millisecond; m/s, meter per second; LRM-1, Logistic regression model-1, and LRM-2, Logistic regression model-2.

^a^ A smaller value indicates a more positive test.

**FIGURE 3:**
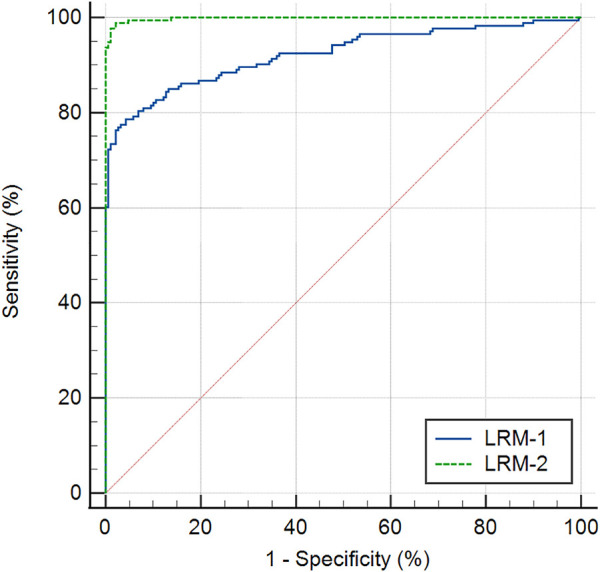
Receiver operating characteristic curves of two multivariable classification models, the Logistic regression model-1 (LRM-1), and Logistic regression model-2 (LRM-2) for keratoconus *versus* the control group.

Table 5 displays two multivariable classification models constructed by Logistic regression analysis. The model LRM-1 (Logistic regression model-1), which comprised of ΔDAR2, ΔIR, and age, obtained the AUROC of 0.922 (95% CI: 0.893–0.952, *p* < 0.001) for identifying keratoconus (including FFKC) (Table 4). Taking ΔBAD-D and ΔARTh into account, another model LRM-2 (Logistic regression model-2), which included ΔIR, Age, ΔARTh, and ΔBAD-D, had the AUROC of 0.998 (95% CI: 0.996–1.000, *p* < 0.001) for identifying keratoconus (including FFKC) (Table 4), which was significantly higher than that of LRM-1 (*z* = 5.108, *p* < 0.001). The corresponding ROC curves are shown in Figure 3.

**TABLE 5:** Logistic regression analysis of bilateral differential parameters in discriminating keratoconus.

	β	S.E.	Wald	df	Sig.	Exp(*β*)
*LRM-1*						
ΔDAR2	2.372	0.598	15.706	1	<0.001	10.717
ΔIR	1.863	0.342	29.645	1	<0.001	6.440
Age	−0.109	0.030	13.072	1	<0.001	0.897
Constant	−0.196	0.766	0.065	1	0.798	0.822
*LRM-2*						
ΔIR	3.723	1.160	10.299	1	0.001	41.385
Age	−0.184	0.078	5.595	1	0.018	0.832
ΔARTh	0.024	0.007	12.630	1	<0.001	1.024
ΔBAD-D	4.798	1.166	16.938	1	<0.001	121.305
Constant	−10.785	3.309	10.623	1	0.001	0.000

Notes: LRM-1, Logistic regression model-1; LRM-2, Logistic regression model-2; ΔDAR2, asymmetry of the deformation amplitude ratio at 2 mm; ΔIR, asymmetry of the integrated radius; ΔARTh, asymmetry of the Ambrosio Relational Thickness horizontal; and ΔBAD-D, asymmetry of the Belin/Ambrosio Enhanced Ectasia Deviation value.

## Discussion

This study compared the characteristics of bilateral differences in corneal biomechanics between the KC and control groups as well as investigated the important role of bilateral biomechanical differences in the early identification of keratoconus and high-risk corneas.

The present study found that most corneal biomechanical parameters, except for CBI, manifested significant bilateral asymmetry in keratoconus when compared with normal eyes, implying that bilateral asymmetry in corneal biomechanics is also a key feature of keratoconus. Moreover, we found that the FFKC cases had similar patterns in corneal tomography when compared with normal eyes, but their corneal biomechanical stability was significantly lower than that of normal eyes (with significantly decreased SP-A1 and increased TBI; Supplementary Table S2). These findings imply that the compromise in corneal biomechanical properties should occur earlier than the changes in corneal tomography, which is consistent with Asroui’s work (Asroui et al., 2022). However, the AUROC of SP-A1 and TBI were too low to meet the requirement of KC screening before corneal refractive surgery. Correlation analysis showed that ΔDAR2 and ΔIR were mildly correlated with FKmax (worse), indicating significant correlations between bilateral asymmetry in biomechanical features and severity of keratoconus. A previous study suggested that the bilateral asymmetry of corneal tomography increases significantly with disease severity (Nichols, 2004; Naderan et al., 2017), which is consistent with the findings of the present study. Herber et al. (2019) revealed that IR and DAR2 showed significant differences among keratoconus groups with different TKC stages. This suggests that the ΔDAR2 and ΔIR may be more sensitive to subtle changes in corneal biomechanics during the development of keratoconus, and would be helpful for the early detection and monitoring of keratoconus.

This study showed that the bilateral differential biomechanical parameters, including ΔDAR2, ΔIR, ΔSP-A1, and ΔMax ICR, had relatively good discriminative ability for keratoconus (Table 4). Previous studies have confirmed the excellent ability of the abovementioned parameters for detecting clinical keratoconus in monocular analysis, and the AUROC reached 0.95 (Chan et al., 2018; Sedaghat et al., 2018; Herber et al., 2019). However, the accuracy decreases significantly in early keratoconus and the AUROC of SP-A1 was 0.762 in screening subclinical keratoconus (Kataria et al., 2019). In the present study, subclinical and forme fruste keratoconus were included in the KC group; therefore, the discriminative accuracies of these parameters were limited in the monocular analysis (Supplementary Table S1), especially for FFKC (Supplementary Table S2), which were consistent with previous findings (Kataria et al., 2019).

Vinciguerra et al. (2016) developed the CBI model based on pachymetric progression and biomechanical characteristics of the cornea, while Ambrosio et al. (2017) developed the TBI model with combined tomographic and biomechanical characteristics. The accuracy of CBI and TBI for detecting clinical keratoconus was close to 100% (Ambrosio et al., 2017) but dropped to 0.684 and 0.884 for screening subclinical keratoconus, respectively (Asroui et al., 2022). In this study, the AUROCs of CBI and TBI were 0.823 and 0.905, respectively (Supplementary Table S1), similar to those of previous studies. However, CBI was not significantly different between the FFKC and control groups, and the discriminative ability of TBI for FFKC was inadequate, with an AUROC of 0.694 (Supplementary Table S2).

However, it is interesting that in the bilateral differential analysis of the present study, the AUROC of ΔDAR2, ΔIR, and ΔMax ICR increased to 0.889, 0.884, and 0.805, respectively, suggesting that the biomechanical differential parameters had good accuracy in screening keratoconus and may be an effective supplement to current screening models for keratoconus based on monocular analysis. Additionally, the logistic regression model LRM-1 was also able to discriminate keratoconus, with an AUROC of 0.922. After including ΔBAD-D and ΔARTh in the analysis, the logistic regression model LRM-2 showed a significantly enhanced ability to identify keratoconus, with an AUROC of 0.998 (Tables 4, 5).

The LRM-1 in this study was composed of ΔDAR2, ΔIR, and age, which combined the bilateral differential characteristics of corneal biomechanics and obtained a better discriminative accuracy than CBI. Another model LRM-2, which consists of ΔIR, age, ΔARTh, and ΔBAD-D, reflected the bilateral differential characteristics of corneal tomography and biomechanics and had excellent accuracy in identifying keratoconus. Our findings demonstrated that the combination of differential biomechanical parameters can achieve better accuracy for screening keratoconus, while the combination of differential tomographic and biomechanical parameters can further improve the sensitivity and specificity. In the future, evaluation of bilateral asymmetry of keratoconus through large samples, multi-modality, and multi-parameters is expected to develop models with greater discriminative abilities. In the bilateral differential analysis, there were no significant differences in ΔCBI and ΔTBI between the KC and control groups. A possible reason may be that both CBI and TBI values are indices for screening rather than normally distributed variables. In binocular keratoconus cases, the values of CBI and TBI commonly increase to 1.0; thus, ΔCBI and ΔTBI may drop to 0. (Supplementary Figures S2G, S2H). Therefore, the values of ΔCBI and ΔTBI cannot precisely reflect the characteristics of bilateral asymmetry in keratoconus.

Moreover, age was considered an important variable in the regression models and was negatively correlated with the risk of keratoconus (*β* = −0.109 for LRM-1 and −0.184 for LRM-2, respectively) (Table 5). Previous studies reported that keratoconus has the most significant incidence in the age range of 20–30 years (Gomes et al., 2022) and that age at presentation was the most significant predictor of progression risk (Maile et al., 2022), which indicated that patients with younger age should be given more attention in the screening and monitoring of keratoconus, in accordance with the present study. Therefore, our findings demonstrated that age should also be regarded as an important factor in assessing at-risk corneas prior to refractive surgery.

We also investigated SSI, an index that reflects the material stiffness of the cornea *in vivo* (Eliasy et al., 2019). Our findings showed a relatively low accuracy of SSI and ΔSSI in screening keratoconus, and had AUROCs of 0.718 and 0.767, respectively. A previous study revealed that the SSI was not significantly different between eyes with mild keratoconus and normal eyes, and the AUROC was 0.642 for detecting keratoconus (Herber et al., 2022), which is similar to that in our study (Supplementary Tables S1, S2), suggesting that the SSI alone was not suitable for screening keratoconus. However, the SSI was proven to decrease significantly during the reduction of corneal stiffness in the progression of keratoconus (Padmanabhan et al., 2022) and increased after corneal cross-linking (Nishida et al., 2022), demonstrating that the SSI may be more suitable for monitoring the progression and treatment efficacy in keratoconus, however, its application in screening keratoconus still needs to be further explored.

This study has several limitations. First, the sample size was relatively small, and detailed subgroup analyses were not performed to distinguish the differential biomechanical characteristics among different types of keratoconus. Second, the Corvis ST merely measures the overall biomechanical properties of the cornea in the horizontal meridian, while the cone of keratoconus is often located in the vertical direction. Therefore, there are defects when using the Corvis ST to measure the corneal biomechanics in keratoconus. Further investigations with larger sample sizes and novel instruments, such as Brillouin microscopy (Zhang et al., 2023) and optical coherence elastography (Kirby et al., 2017), are required. Third, while the significantly increased bilateral asymmetry of corneal tomography or biomechanics would be helpful for the early identification of keratoconus, it does not necessarily imply that the increased bilateral asymmetry is keratoconus. The presence of inter-eye asymmetry suggests that ophthalmologists should actively search for potential causes that lead to inter-eye asymmetry, such as history of trauma, oculopathy, or surgeries. Failure to identify a clear cause may suggest abnormal biomechanical attenuation in the worse eye, which could result in a higher risk of corneal ectasia after refractive surgery than in the general population. For such patients, further observations and thorough notifications of surgical risks are absolutely necessary.

In summary, the bilateral differences in corneal biomechanical parameters, including ΔDAR2, ΔIR, ΔSP-A1, and ΔMax ICR, were significantly increased in KC compared with those in the control group and may be helpful for the early screening of keratoconus. The Logistic regression model combining bilateral differential characteristics of corneal biomechanics and corneal tomography is expected to improve the discriminative efficacy for early keratoconus.

## Data Availability

The raw data supporting the conclusion of this article will be made available by the authors, without undue reservation.
