# Supplementary material for: Comparison of bilateral differential characteristics of corneal biomechanics between keratoconus and normal eyes
Source: Front Bioeng Biotechnol. 2023 Jun 1;11:1163223. doi: 10.3389/fbioe.2023.1163223 (PMC10267412; doi:10.3389/fbioe.2023.1163223)
Supplement: Supplementary file 1 [file DataSheet1.PDF]

Supporting Informations

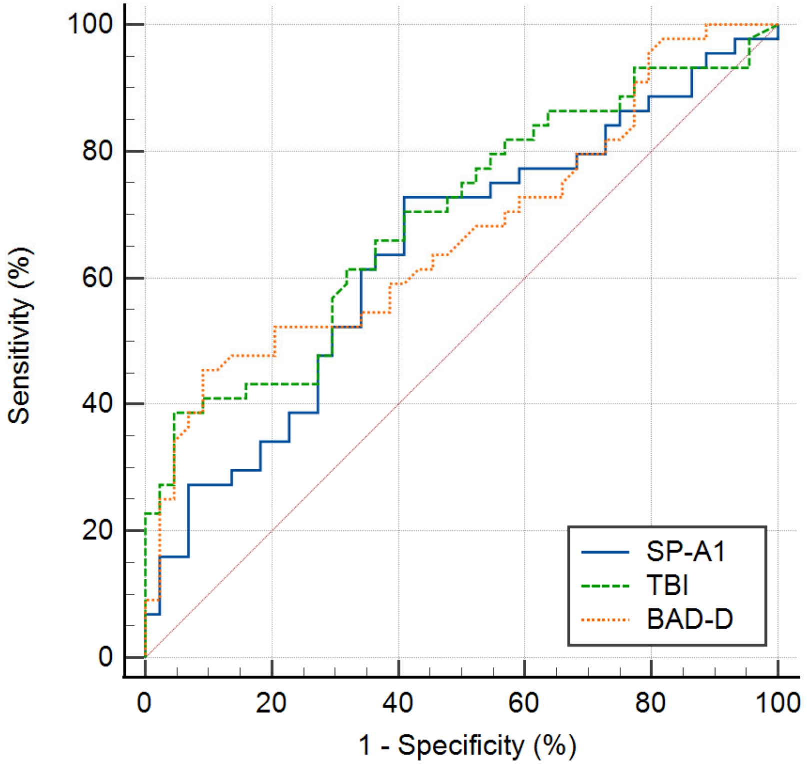

Supplementary Figure 1. Receiver operating characteristic curves of major biomechanical and topographic parameters for discriminating forme fruste keratoconus. (SP-A1: the stiffness parameter at the first applanation; TBI: the Tomographic and Biomechanical Index; BAD-D: the Belin/Ambrosio Enhanced Ectasia Deviation value.)

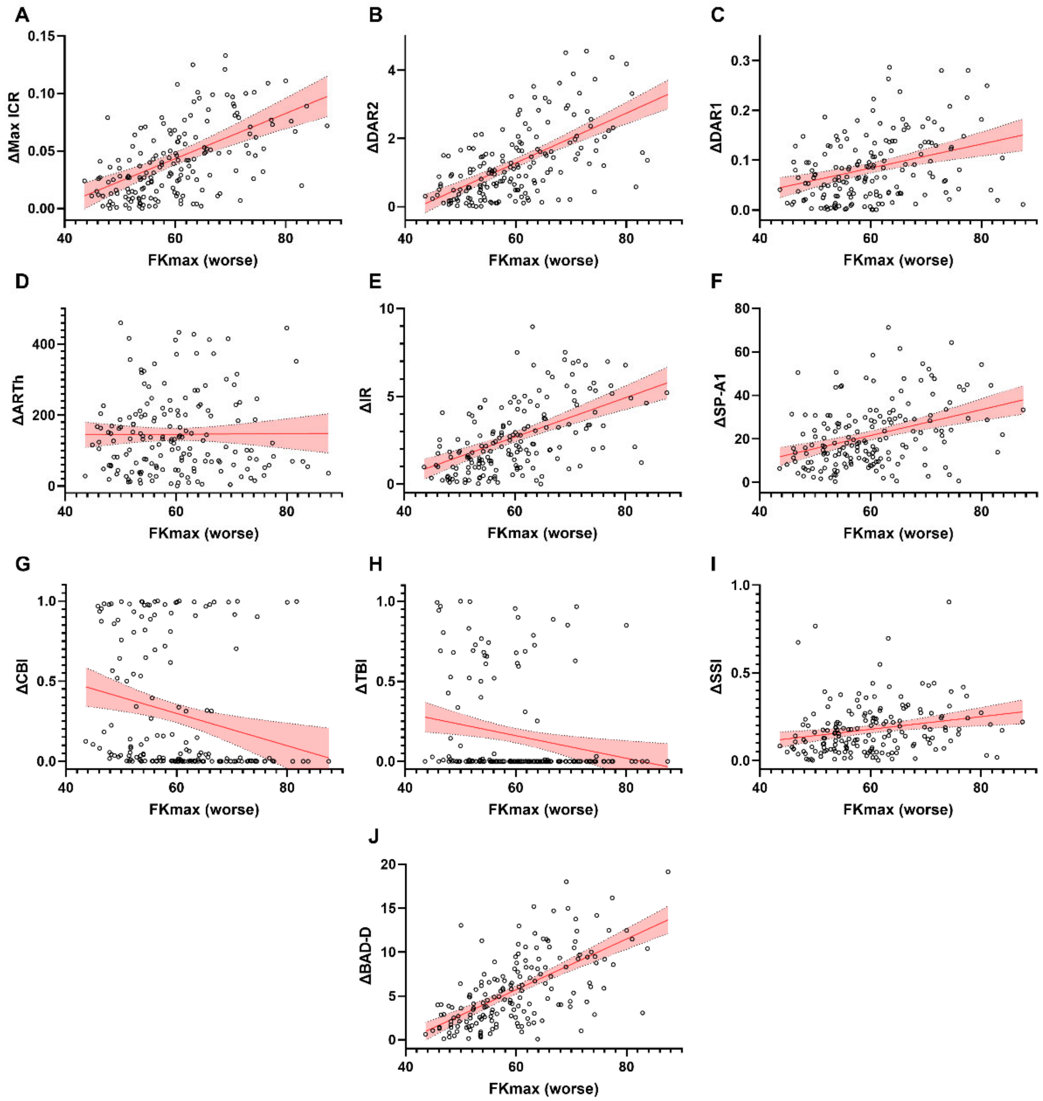

Supplementary Figure 2. Relationship between FKmax(worse) and major bilateral differential parameters.The red lines represented the simple linear regression of corresponding parameters and the 95% confidence interval. (FKmax(worse): the maximum keratometry of the anterior surface of the "worse" eye; ΔMax ICR: asymmetry of the maximum inverse concave radius; ΔDAR2: asymmetry of the deformation amplitude ratio at 2mm; ΔDAR1: asymmetry of the deformation amplitude ratio at 1mm; ΔARTh: asymmetry of the Ambrosio Relational Thickness horizontal; ΔIR: asymmetry of the integrated radius; ΔSP-A1: asymmetry of

the stiffness parameter at the first applanation; ΔCBI: asymmetry of the Corvis Biomechanical Index; ΔTBI: asymmetry of the Tomographic and Biomechanical Index; ΔSSI: asymmetry of the stress-strain index; ΔBAD-D: asymmetry of the Belin/Ambrosio Enhanced Ectasia Deviation value.)

Supplementary Table 1. Receiver operating characteristics analysis for corneal biomechanical parameters in discriminating keratoconus

Parameters	AUROC	*P*	95% CI	Cut-off	Sensitivity	Specificity
A1V (m/s)	0.751	0.000	0.701 to 0.802	0.16	53.2%	88.9%
A2V (m/s)	0.684	0.000	0.627 to 0.74	-0.28 #	51.4%	83.1%
A1DeflA (mm)	0.755	0.000	0.702 to 0.807	0.10	53.2%	93.7%
A2DeflA (mm)	0.659	0.000	0.602 to 0.716	0.11	47.4%	81.0%
DeflA Max (mm)	0.718	0.000	0.665 to 0.771	0.93	62.4%	71.4%
Max ICR (mm^{-1})	0.753	0.000	0.7 to 0.805	0.21	54.3%	93.1%
DAR2	0.792	0.000	0.745 to 0.839	5.19	52.0%	96.3%
DAR1	0.780	0.000	0.731 to 0.829	1.65	54.9%	93.1%
ARTh	**0.818**	0.000	0.772 to 0.865	**388.99** #	**67.6%**	**91.0%**
IR (mm^{-1})	0.779	0.000	0.73 to 0.829	10.33	57.2%	94.2%
SP-A1 (mmHg/mm)	**0.851**	0.000	0.81 to 0.891	**86.60** #	**61.8%**	**95.2%**
CBI	**0.823**	0.000	0.777 to 0.868	**0.66**	**65.9%**	**94.7%**
TBI	**0.905**	0.000	0.87 to 0.939	**0.46**	**82.1%**	**95.8%**
SSI	0.718	0.000	0.664 to 0.772	0.81 #	54.3%	85.2%
BAD-D	**0.900**	0.000	0.865 to 0.935	**2.09**	**77.5%**	**97.4%**

Notes: AUROC: area under the curves of receiver operating characteristic; 95% CI: 95% confidence interval for AUROC; Cut-off: the threshold values; A1V: velocity at the first applanation; A2V: velocity at the second applanation; A1DeflA: the deflection amplitude at the first applanation; A2DeflA: the deflection amplitude at the second applanation; DeflA Max: the maximum deflection amplitude; Max ICR: the maximum inverse concave radius; DAR2: the deformation amplitude ratio at 2mm; DAR1: the deformation amplitude ratio at 1mm; ARTh: the Ambrosio Relational Thickness horizontal; IR: integrated radius; SP-A1: the stiffness parameter at the first applanation; CBI: the Corvis Biomechanical Index; TBI: the Tomographic and Biomechanical Index; SSI: the stress-strain index; BAD-D: the Belin/Ambrosio Enhanced Ectasia Deviation value; mmHg/mm: millimeter of mercury per millimeter; mm: millimeter; m/s: meter per second.

a smaller value indicates more positive test.

Supplementary Table 2. Receiver operating characteristics analysis for corneal biomechanical parameters in discriminating forme fruste keratoconus

Parameters	FFKC group (n=44) Mean ± SD	Control group (n=44) Mean ± SD	Z^a	P^a	AUROC	Cut-off	Sensitivity	Specificity
A1V (m/s)	0.15 ± 0.02	0.14 ± 0.02	-0.931	0.352	0.558	0.16	31.8%	90.9%
A2V (m/s)	-0.26 ± 0.04	-0.26 ± 0.02	-0.931	0.352	0.558	-0.25 [#]	47.7%	77.3%
A1DeflA (mm)	0.09 ± 0.01	0.09 ± 0.01	-0.878	0.380	0.554	0.11	22.7%	97.7%
A2DeflA (mm)	0.1 ± 0.02	0.1 ± 0.01	-0.555	0.579	0.534	0.12	25.0%	97.7%
DeflA Max (mm)	0.88 ± 0.11	0.89 ± 0.09	-0.434	0.664	0.527	0.89	54.5%	56.8%
Max ICR (mm^{-1})	0.19 ± 0.02	0.19 ± 0.02	-0.968	0.333	0.560	0.18	54.5%	70.5%
DAR2	4.51 ± 0.49	4.51 ± 0.42	-0.200	0.841	0.512	4.67	72.7%	40.9%
DAR1	1.59 ± 0.05	1.59 ± 0.04	-0.784	0.433	0.549	1.58	47.7%	70.5%
ARTh	485.2 ± 92.87	505.84 ± 91.62	-0.901	0.367	0.556	424.92 [#]	29.5%	86.4%
IR (mm^{-1})	8.87 ± 1.07	8.99 ± 0.96	-0.559	0.576	0.535	9.42	72.7%	40.9%
SP-A1 (mmHg/mm)	98.85 ± 17.19	107.15 ± 16.5	-2.278	**0.023**	**0.641**	**104.23** [#]	**72.7%**	**59.1%**
CBI	0.19 ± 0.31	0.13 ± 0.21	-0.932	0.352	0.558	0.00	84.1%	36.4%
TBI	0.45 ± 0.35	0.21 ± 0.19	-3.134	**0.002**	**0.694**	**0.47**	**38.6%**	**95.5%**
SSI	0.97 ± 0.17	0.93 ± 0.12	-1.072	0.284	0.566	0.95 [#]	54.5%	63.6%
BAD-D	1.56 ± 0.63	1.14 ± 0.53	-2.746	**0.006**	**0.670**	**1.64**	**45.5%**	**90.9%**

Notes: FFKC: forme fruste keratoconus; SD: standard deviation; AUROC: area under the curves of receiver operating characteristic; Sig: significance; Cut-off: the threshold values; A1V: velocity at the first applanation; A2V: velocity at the second applanation; A1DeflA: the deflection amplitude at the first applanation; A2DeflA: the deflection amplitude at the second applanation; DeflA Max: the maximum deflection amplitude; Max ICR: the maximum inverse concave radius; DAR2: the deformation amplitude ratio at 2mm; DAR1: the deformation amplitude ratio at 1mm; ARTh: the Ambrosio Relational Thickness horizontal; IR: integrated radius; SP-A1: the stiffness parameter at the first applanation; CBI: the Corvis Biomechanical Index; TBI: the Tomographic and Biomechanical Index; SSI: the stress-strain index; BAD-D: the Belin/Ambrosio Enhanced Ectasia Deviation value; mmHg/mm: millimeter of mercury per millimeter; mm: millimeter; m/s: meter per second.

[#] a smaller value indicates more positive test; [a] Mann-Whitney test.
